# Changes in Antioxidant Properties and Amounts of Bioactive Compounds during Simulated In Vitro Digestion of Wheat Bread Enriched with Plant Extracts

**DOI:** 10.3390/molecules26206292

**Published:** 2021-10-18

**Authors:** Anna Czubaszek, Anna Czaja, Anna Sokół-Łętowska, Joanna Kolniak-Ostek, Alicja Z. Kucharska

**Affiliations:** 1 Department of Fermentation and Cereals Technology, Wrocław University of Environmental and Life Sciences, 51-630 Wroclaw, Poland; anna.czubaszek@upwr.edu.pl (A.C.); anna.czaja@upwr.edu.pl (A.C.); 2 Department of Fruit, Vegetable and Plant Nutraceutical Technology, Wrocław University of Environmental and Life Sciences, 51-630 Wroclaw, Poland; anna.sokol-letowska@upwr.edu.pl

**Keywords:** in vitro simulated digestion, isoflavones, procyanidins, flavonols, microencapsulation, maltodextrin, inulin, hawthorn bark, soybeans, onion husk

## Abstract

Cereal preparation can be an excellent source of substances with proven health-promoting properties. Unfortunately, some types of bread, such as white flour bread, are devoid of many valuable nutrients. Therefore, it is necessary to look for ways to increase its density and nutritional value. The aim of the study was to investigate the effect of stabilized plant extracts on the quality of bread, its antioxidant activity and polyphenol content, and to evaluate the stability of bioactive compounds and antioxidant activity during in vitro digestion. The research material was the wheat bread baked with spray dried microcapsules of hawthorn bark, soybeans and onion husks in maltodextrin or inulin carriers. The addition of plant extracts resulted in the presence of phenolic compounds in the wheat bread, and its antioxidant activity significantly increased. There was no significant difference in antioxidant activity between breads containing microcapsules with different carriers. During in vitro digestion, procyanidins and isoflavones in bread were more resistant to the digestive processes than other compounds. The antioxidant activity during simulated digestion was the highest at the stage of gastric digestion, and its value depended on the extract used and the analytical method applied.

## 1. Introduction

Bread is an important component of the diet in many parts of the world. Consumers expect it to be of high organoleptic quality, as well as density and nutritional value (corresponding to dietary recommendations). Improving the nutritional value of bread can be achieved by improving the production process or enriching recipes. Compounds with promising potential for application to bread are polyphenols, which are characterized by a multitude of types and configurations of substituents [1]. Among these compounds, there are such subgroups as flavonoids (including anthocyanins, flavonols, flavan-3-oils, isoflavones), phenolic acids and others. The main source of polyphenols is raw materials of plant origin, and different phenolic compound profiles exist in different plants. For example, flavonols are present in onions [2], isoflavones are found in soybeans [3] and hawthorn is a rich source of procyanidins [4].

Some of the health-promoting polyphenols are found in plants with limited application possibilities, in non-edible plant parts or in waste products in processing. The extraction process can solve the problems related to the limited application possibilities of these compounds. An example of such a procedure is the use of extracts obtained from various parts of hawthorn [4,5] or the use of onion husk, which is a waste in various branches of the food industry and is a valuable source of flavonols [6].

Polyphenols exhibit a broad spectrum of biological functions and biochemical activity. It has been proven that they have a beneficial effect on human health and the efficiency of the cardiovascular system [7] and lower blood cholesterol levels [8]. They also have high antioxidant potential and high bioactivity of metabolites [9]. The disadvantageous feature of polyphenols is their considerable susceptibility to external factors. For this reason, their content during production processes and food preparation decreases under the influence of various types of operations. They show instability in the event of changes in parameters such as pH [10], temperature [11,12], access to light [13] and exposure to various environmental factors [14,15]. Their content has been shown to decrease during thermal processing (cooking, microwave, steaming, baking) [16,17]. The amount of polyphenols in food is also influenced by the time and conditions of its storage [18,19,20]. Literature data also indicate that these compounds are sensitive to conditions in the digestive tract and in the process of simulated in vitro digestion [21,22,23,24]. The bioavailability of polyphenols from the diet is shaped by many factors, including: external environmental factors (degree of maturity, sun exposure), factors related to food processing (temperature, homogenization, freeze-drying, cooking / processing, storage), factors related to structure of polyphenols (chemical structure), factors related to the work of the intestines (enzyme activity, intestinal passage, colon microflora), systemic factors (age and sex, diseases and/or pathologies, genetic factors), factors related to food (type of product, presence of positive or negative components affecting absorption, such as fat and fiber) and interactions with other compounds [21,25,26,27].

Taking into account the sensitivity of polyphenols to external factors and possible adverse interactions with various ingredients of food products, methods are sought to improve their chemical stability and bioavailability and reduce their sometimes-negative impact on the organoleptic properties of baking. An example of a technology enabling the protection of these compounds against the adverse effects of the environment, food production processes and digestive processes is microencapsulation. This process may also contribute to the potential reduction of their negative impact on the organoleptic properties of the finished product [28]. In addition, the ingredients used as the material constituting the shell or the matrix structure, in addition to protecting the active compounds and modulating their action, can modify the properties of microcapsules in food products and even affect the properties of food products. It has been shown that inulin, used as a carrier in microcapsules, has a health-promoting effect [29], as well as extending the stability of the dough and reducing its softening during mechanical processing, increases the bread yield and hardness of the bread crumb, and reduces the loaf volume, which also causes the bread to be darker in color compared to the control bread [30,31,32]. Moreover, maltodextrins, used as a carrier, affect the baking properties of flour: they strengthen the dough for mechanical processing, increase the volume of the bread [33], improve the texture of the crust during storage and contribute to a darker color of the bread [34,35].

The literature indicates that research is being undertaken on the preparation of polyphenolic compounds by various methods, their technological properties, antioxidant activity and stability. However, there are few reports on the use of such additives for bread enrichment and the bioavailability of these compounds. For this reason, research was undertaken to determine the quality of bread with the addition of extracts from the bark of young hawthorn shoots, soybeans and onion husks microencapsulated in inulin and maltodextrin, and to evaluate the stability of phenolic compounds derived from these extracts during in vitro digestion of bread with their addition.

## 2. Results and Discussion

### 2.1. Characteristics of Wheat Bread

#### 2.1.1. Color, Yield and Volume of Bread

**Figure 1:**
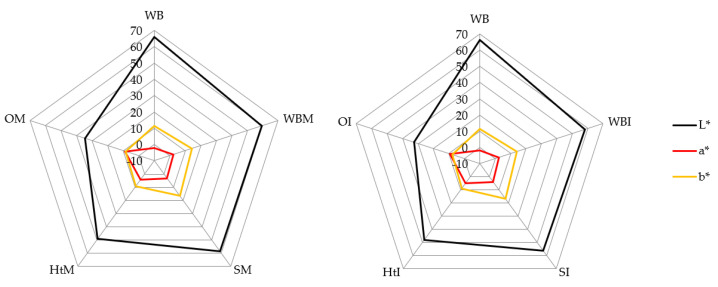
Color of different samples of wheat bread. Abbreviations: WB—wheat bread; WBM—wheat bread with maltodextrin; SM—wheat bread enriched with soy extracts in maltodextrin; HtM—wheat bread enriched with hawthorn extracts in maltodextrin; OM—wheat bread enriched with onion husk extracts in maltodextrin; WBI—wheat bread with inulin; SI—wheat bread enriched with soy extracts in inulin; HtI—wheat bread enriched with hawthorn extracts in inulin; OI—wheat bread enriched with onion husk extracts in inulin. The data are expressed as mean (*n* = 3).

The effect of the addition of extracts of hawthorn bark, soybeans and onion husks microencapsulated in inulin and maltodextrin in the proportion 1:3 on the quality and organoleptic characteristics of bread was assessed. Figure 1 and Table S1 show the average values of the L*, a* and b* color parameters.

It was found that the addition of inulin and maltodextrin decreased the brightness of the bread (L* = 66.20 for wheat bread (WB), 59.60 for wheat bread maltodextrin (WBM) and 58.40 for wheat bread inulin (WBI)). Some authors [34,35] report that the use of maltodextrin as a bread additive causes darkening of the bread crumb. Presumably, this may be due to the increased intensity of the Maillard reaction, involving maltodextrin, occurring during baking [36]. Among the breads containing plant extracts, bread with onion husk extract (L* = 32.6 for OM and 34.7 for OI) was the darkest and bread with soy microcapsules (L* = 58.80 for SM and 56.5 for SI) was the brightest, which is consistent with the results of studies by other researchers [37]. Moreover, Czaja et al. [18], Wang et al. [38] and Gawlik-Dziki et al. [39] observed that the addition of plant extracts changes the color of the bread. Negative values of the a* parameter were obtained only in wheat bread (WB), for which a* = −2.00. It was also observed that the addition of extracts increased the proportion of red color in the tested breads. In the case of the breads with the addition of extracts, an increase in the yellow color was observed, compared to the wheat bread (WB), for which b* = 11.30. The greatest proportion of this color was found in breads containing soybean extract—b* = 16.70 for SM and 17.00 for SI. Bread containing preparations of soybean extracts, onion husks and hawthorn bark had a color typical of conventional bread, because the color of compounds obtained from these plants is in a color palette similar to bread [40].

**Figure 2:**
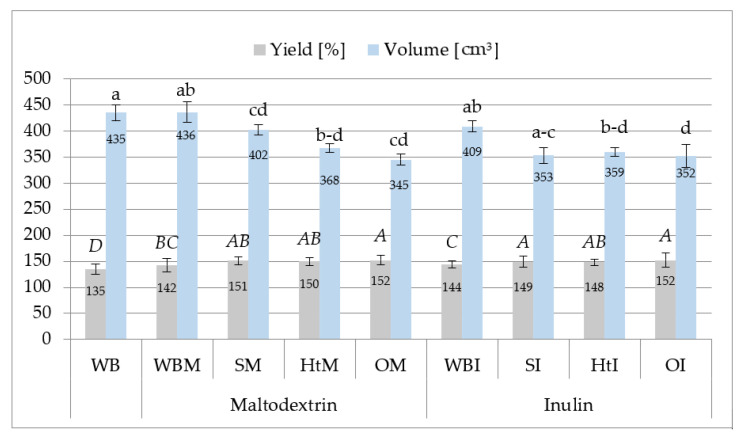
Yield (%) and volume (cm^3^) of different samples of wheat bread. Abbreviations: WB—wheat bread; WBM—wheat bread with maltodextrin; SM—wheat bread enriched with soy extracts in maltodextrin; HtM—wheat bread enriched with hawthorn extracts in maltodextrin; OM —wheat bread enriched with onion husk extracts in maltodextrin; WBI—wheat bread with inulin; SI—wheat bread enriched with soy extracts in inulin; HtI—wheat bread enriched with hawthorn extracts in inulin; OI—wheat bread enriched with onion husk extracts in inulin. Values with different letters were significantly different at *p* = 0.05 according to Duncan’s test. The data are expressed as mean (*n* = 3).

The characteristics of wheat bread and its quality largely depend on the quality of the flour and the recipe ingredients. Even if wheat flour has a very good baking value, by introducing various additives into the recipe, including those aimed at increasing the nutritional value of bread, changes in the quality of the finished product should be expected due to the imbalance in the gluten cross-linked structure caused by the added ingredient [41]. One of the distinguishing features of the bread quality is its volume. Figure 2 shows the average volumes of wheat bread with and without plant extracts.

The results show that the source of the extract and the carrier used had an effect on the volume of bread. The volume was lower than that of the control wheat bread, for all breads containing plant extracts, regardless of the carrier used. The exception was wheat bread with the addition of maltodextrin (WBM), the volume of which was 436 cm^3^. Karolini-Skaradzińska et al. [33] found a positive effect of the addition of maltodextrin on the volume of bread, and Morris and Morris [32] indicated that inulin reduces the volume of bread. The research by Michalak-Majewska et al. [40] indicated that the addition of powdered dried husks from various onion varieties to the recipe of wheat bread in the amount of 2.5% reduces the baking volume. Other studies [42] have shown that a 3% addition of plant extracts in the presence of highly methylated pectins reduces the volume of bread, regardless of the source of the extract and the type of compounds in the extract. In earlier studies by Czaja et al. [18], no changes in the volume of bread were found under the influence of 0.5% and 1.0% of the addition of dried onion extract.

The second important quality factor of bread for technological reasons is its yield. The average values of the yield of wheat bread with and without plant extracts are shown in Figure 2. The yield of wheat bread (WB) was high: 135%. It was found that the increase in this parameter as compared to the control bread was caused by all the additives used. The highest yield of bread was noted for bread with onion extracts (152% for OM and OI), while the lowest was for bread maltodextrin (WBM) and inulin (WBI)—142 and 144%, respectively. Comparing the effect of the addition of plant extracts fixed in inulin or maltodextrin on the qualitative features of wheat bread, it was found that the additives used did not differentiate this feature.

#### 2.1.2. Phenolic Concentration in Bread

**Figure 3:**
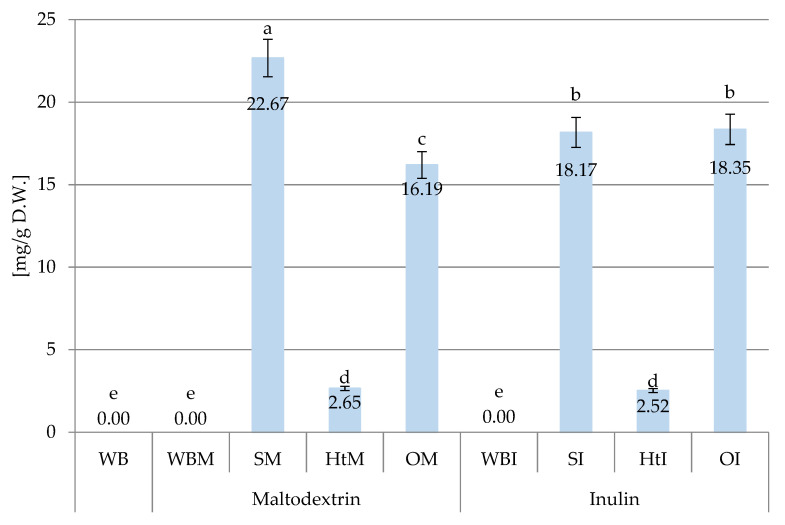
Sum o polyphenolic compounds of different samples of wheat bread. Abbreviations: WB—wheat bread; WBM—wheat bread with maltodextrin; SM—wheat bread enriched with soy extracts in maltodextrin; HtM—wheat bread enriched with hawthorn extracts in maltodextrin; OM—wheat bread enriched with onion husk extracts in maltodextrin; WBI—wheat bread with inulin; SI—wheat bread enriched with soy extracts in inulin; HtI—wheat bread enriched with hawthorn extracts in inulin; OI—wheat bread enriched with onion husk extracts in inulin. Values with different letters were significantly different at *p* = 0.05 according to Duncan’s test. The data are expressed as mean (*n* = 3).

Figure 3 and Tables S2–S4 (in the Supplementary Materials), show the content of polyphenolic compounds in wheat bread (WB), with the addition of maltodextrin (WBM) and inulin (WBI) and with the addition of soybean extracts (SM, SI), hawthorn (HtM, HtI) and onion husks (OM, OI) encapsulated in maltodextrin and inulin.

No polyphenolic compounds were found either in the bread without additives or in the bread with the addition of inulin and maltodextrin (Figure 3). The addition of plant extracts contributed to the presence of polyphenols in the bread. The highest content of polyphenolic compounds (22.67 mg/g dry weight [DW]) was found in bread with the addition of soy in maltodextrin (SM). The smallest amount of bioactive compounds was introduced with the hawthorn extract (2.65 and 2.52 mg/g DW, respectively in maltodextrin and inulin encapsulated).

Many researchers [39,43,44] have shown that various raw materials and products rich in polyphenolic compounds introduced into the recipe increase the content of these compounds in bread. Lee and Cho [45] and Prabhakaran et al. [46] studied the content of isoflavones in soybeans and in a soybean extract preparation. The authors stated that genistein and daidzein constitute the largest proportion of soybean isoflavones. Similarly, in the extracts used in our research, the listed compounds, out of the nine identified ones, were present in the highest amounts (Table S2). The same nine compounds were also identified in bread containing microcapsules with soybean extract. In the SM bread, daidzein (4.39 mg/g DW) and malonylgenistin and genistein (3.85 and 3.84 mg/g DW, respectively) were the dominant polyphenol compounds, while in SI bread it was genistein and daidzein (8.11 and 4.60 mg/g DW, respectively).

Breads containing hawthorn bark microcapsules in inulin or maltodextrin contained procyanidins, (–)-epicatechin and di-, tri- and tetramers, in total 8 compounds (Table S3). Hawthorn bark microcapsules, in addition to the compounds identified in bread, also contained procyanidin B4. Włoch et al. [5] identified 18 compounds in hawthorn bark, i.e., 13 flavan-3-ols, 2 flavonols and 3 flavone derivatives. The dominant group was flavan-3-ols, which included a monomer ((–)-epicatechin), dimers (e.g., dimer B2), trimers (e.g., trimer C1) and tetramers. In the breads with the addition of hawthorn, both for microcapsules obtained with maltodextrin and inulin, the dominant compound was procyanidin C1—1.13 and 1.17 mg/g DW, respectively (Table S3).

Breads with the addition of onion microcapsules were characterized by a high content of quercetin—17.59 mg/g DW for SM and 15.65 mg/g DW for SI (Table S4). Of the six compounds identified in the onion microcapsules, quercetin 4-glucoside was also present in the bread. In previous studies [18], only these two compounds were found in breads enriched with onion extract, and it was found that their content changed slightly during 72 h of storage. The storage temperature also had no significant effect. In our own research, contrary to the reports of other authors [47,48], no quercetin diglucosides were identified in the bread, presumably because only small amounts of 7,4- and 3,4-diglucoside of quercetin were found in the microcapsules used in our research.

Araujo-Díaz et al. [49] proved that maltodextrin is a more effective carrier than inulin because in the samples of the microencapsulated blueberry extract in maltodextrin, more polyphenols were identified than in the microcapsules of this extract with inulin. Bąkowska-Barczak and Kołodziejczyk [50] believe that the stability of polyphenols protected in microcapsules is influenced by the degree of saccharification of carbohydrate carriers—the lower the degree of saccharification, the higher the content of polyphenols. This may be associated with less reactivity and therefore greater stability of the carrier molecules. However, the studies by Ersus and Yurdagel [51] and Tolun, Altintas and Artik [52] did not confirm this hypothesis, because the content of flavonoids in the samples studied by these authors was lower when using a low-sugar maltodextrin extract as a carrier compared to samples with high-sugar maltodextrin or gum Arabic. In the present research, maltodextrin was a better carrier than inulin for soybean extract, and the opposite relationship was found for onion extract. Only for the hawthorn extract were no significant differences found.

#### 2.1.3. Antioxidant Capacity in Bread

**Figure 4:**
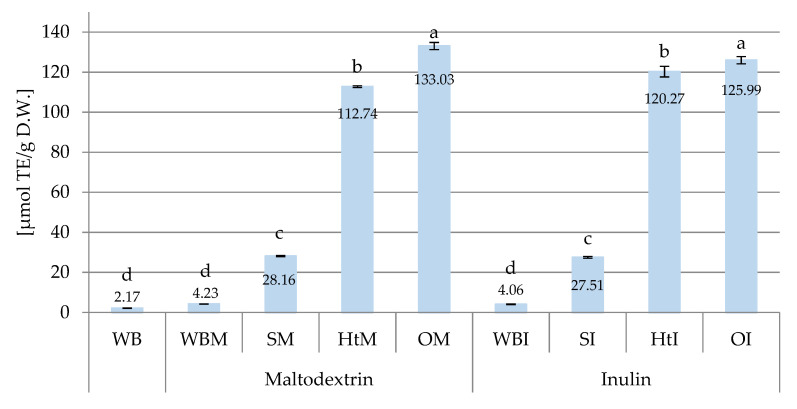
ABTS antioxidant capacity (μmol TE/g D.W.) of different samples of wheat bread. Abbreviations: WB—wheat bread; WBM—wheat bread with maltodextrin; SM—wheat bread enriched with soy extracts in maltodextrin; HtM—wheat bread enriched with hawthorn extracts in maltodextrin; OM—wheat bread enriched with onion husk extracts in maltodextrin; WBI—wheat bread with inulin; SI—wheat bread enriched with soy extracts in inulin; HtI—wheat bread enriched with hawthorn extracts in inulin; OI—wheat bread enriched with onion husk extracts in inulin. Values with different letters were significantly different at *p* = 0.05 according to Duncan’s test. The data are expressed as mean (*n* = 3).

**Figure 5:**
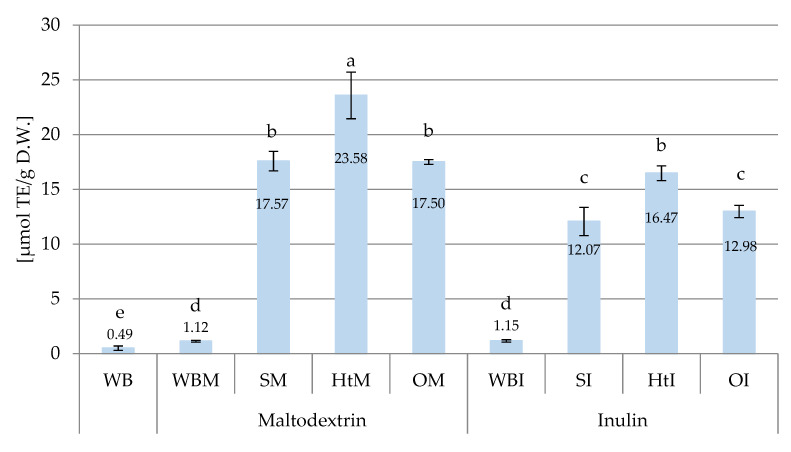
FRAP antioxidant capacity (μmol TE/g D.W.) of different samples of wheat bread. Abbreviations: WB—wheat bread; WBM—wheat bread with maltodextrin; SM—wheat bread enriched with soy extracts in maltodextrin; HtM—wheat bread enriched with hawthorn extracts in maltodextrin; OM—wheat bread enriched with onion husk extracts in maltodextrin; WBI—wheat bread with inulin; SI—wheat bread enriched with soy extracts in inulin; HtI—wheat bread enriched with hawthorn extracts in inulin; OI—wheat bread enriched with onion husk extracts in inulin. Values with different letters were significantly different at *p* = 0.05 according to Duncan’s test. The data are expressed as mean (*n* = 3).

Figure 4 and Figure 5 show the antioxidant activity determined before digestion in breads with the addition of inulin, maltodextrin and microcapsules containing polyphenolic compounds of hawthorn, soybean and onion using ABTS^+•^ and FRAP methods, respectively.

The obtained results indicate that the antioxidant activity of wheat bread determined by the ABTS^+•^ method was very low at 2.17 µmol TE/g DW (Figure 4). The addition of 3% of inulin or maltodextrin to wheat bread resulted in a statistically insignificant but almost twofold increase in antioxidant activity (4.06 µmol for WBI and 4.23 µmol TE/g DW for WBM). The addition of microencapsulated plant extracts significantly increased the antioxidant activity in bread. In breads with onion husk extract (133.02 µmol TE/g DW—OM and 125.99 µmol TE/g DW—OI) and hawthorn bark (112.74 µmol TE/g DW—HtM and 120.27 µmol TE/g DW—HtI) it was more than thirty times in relation to bread containing only extract carriers and about sixty times in relation to wheat bread. It follows that the source of antioxidant activity is the extracts, and the carriers have modulating and technological properties. Of the breads enriched with extracts, a significantly lower level of ABTS^+•^ cation inactivation was found in breads with the addition of microencapsulated SM and SI soybean extracts (28.16 and 27.51 µmol TE/g DW, respectively).

The values of the results obtained with the FRAP method were lower than those with the ABTS^+•^ method (Figure 5). In wheat (WB) and maltodextrin (WBM) or inulin (WBI) bread, the results were four times lower, and in those with SI and SM extract, the results were half the values obtained in the ABTS^+•^ method. In the remaining tested samples, the activity was almost ten times lower than in the ABTS^+•^ method. The highest FRAP values were found in breads with the addition of hawthorn bark extracts (HtM—23.58 µmol Trolox/g DW and HtI—16.47 µmol Trolox/g DW), and the ability to reduce Fe^2+^ ions in breads containing SM and SI was similar to those in breads with the addition of OM and OI (Figure 5).

The increase in antioxidant activity observed in our research in breads with the addition of microencapsulated extracts of hawthorn bark, soybean seed and onion husk extracts is in line with literature reports that indicate that the antioxidant activity (measured by any method) increases with the addition of plant extract [39,40,53]. We found that the antioxidant capacity of bread depended on the type of plant from which the extract was obtained, introduced into the recipe. This means that the type of biologically active compounds and the source of the extract used were an important factor shaping the antioxidant properties of the bread. The dependence of the antioxidant activity of compounds present in plants on the species or even variety within one species has been observed by many researchers. This phenomenon has been described in studies on fruit [12,54], vegetables [16] and herbs and spices [55].

The results in Figure 5 show that encapsulation with maltodextrin was more advantageous, because the breads containing the extracts fixed on this carrier had significantly higher FRAP values than those containing microcapsules with inulin. A similar tendency can be noted among the results obtained with the ABTS^+•^ method in breads with SM and SI, as well as OM and OI (Figure 4). Araujo-Díaz et al. [49] demonstrated the high effectiveness of maltodextrin in protecting the active compounds of blueberry juice, which was greater than the effectiveness of inulin. Lacerda et al. [56], in addition to confirming the beneficial effect of maltodextrin on the activity of microcapsules, also observed that the level of antioxidant activity measured by the FRAP method was correlated with the content of anthocyanins in microcapsules. Other literature reports also indicate maltodextrin as a more suitable extract carrier than inulin [57].

### 2.2. Bioavailability of Bioactive Compounds

#### 2.2.1. Bioactive Compounds

The content of phenolic compounds in liquids collected after successive digestion stages of the tested breads enriched with plant extracts was determined by the HPLC method and is presented in Table 1, Table 2 and Table 3. In vitro digestion took place in 3 stages corresponding to digestion in the oral cavity (stage I), stomach (stage II) and intestine (stage III). The activity of the compounds absorbed during digestion was determined in samples taken from the membrane (stage IV).

Table 1 shows the results obtained during in vitro digestion of wheat bread with the addition of 4% microencapsulated soybean extract (SM, SI).

In the samples before digestion, compounds identified from the group of isoflavones were daidzin, glycitin, genistin, daidzein and genistein and their derivatives: malonyldaidzin, malonylglycitin, malonylgenistin isomer and malonylgenistin (Table S2). In consecutive stages of the simulated in vitro digestion, the content of isoflavones was successively reduced. Moreover, after simulated digestion with saliva (stage I), the glycitin and daidzein identified in the bread were decomposed. Wyspiańska et al. [58] carried out in vitro digestion of an isotonic drink enriched with microcapsules of isoflavones with maltodextrin and inulin. At each stage of digestion, the authors identified isoflavones in different amounts, depending on the structure of the active compounds and the carrier. Studies on rats have shown that daidzein and genistein are absorbed in the gastrointestinal tract, while their glycosides are not absorbed [27]. On the other hand, Rodríguez-Roque et al. [26], who subjected soy milk to in vitro digestion, observed an increase in the content of isoflavones at individual stages of digestion. The mentioned compounds in this study were detectable up to the last stage of digestion and glucosides were characterized by higher bioavailability. The cited study [9] also assessed changes in isoflavones during digestion of mixtures of soy milk with juices of various fruits (orange, kiwi, pineapple). In this experiment, isoflavones were also detected at each stage of digestion, similarly to results for soy milk without additives, but their content decreased in the successive stages of digestion, and aglycones were characterized by greater bioavailability. The results of this study suggest that the environment in which they are located has a large impact on the availability and stability of flavone compounds; in this case, the pH of the mixtures was probably an important factor.

The breads with the addition of microencapsulated soybean extract with inulin as a carrier had a higher content of isoflavones before digestion than breads with microencapsulated maltodextrin (Table S2). It was different in isotonic drinks enriched with microcapsules of isoflavones with maltodextrin and inulin [58]. The authors found more isoflavones in beverages with maltodextrin capsules than with inulin. This may be explained by a different product matrix (drink–liquid) than in this study (bread–solid). In the liquids at the individual steps of digestion, the content of the compounds identified in the breads containing microcapsules with inulin and maltodextrin was similar (Table 1). It was also found that during the digestion of bread with the extract fixed in maltodextrin, the tested samples had more daidzin, genistin and malonylgenistin than those in which microcapsules with inulin were used. Differences in the content of this compound in samples with different extract carriers at the same digestion steps may prove that the carriers affect the bioavailability of the compounds derived from the extracts.

Table 2 shows the results obtained during the in vitro digestion of wheat bread with the addition of 4% microencapsulated hawthorn extract (HtM, HtI).

In the samples before digestion compounds from the group of procyanidins were identified: dimer (B5), procyanidin B2, (−)-epicatechin, procyanidin C1, three tetramers and a trimer (Table S3). It was found that in the consecutive stages of the simulated in vitro digestion, the content of procyanidins was successively decreased. Moreover, during digestion, some of the compounds identified in bread decomposed before digestion, and at the third stage, after simulated intestinal digestion, only two compounds were identified: (−)-epicatechin and procyanidin C1 and only (−)-epicatechin in the membrane. Moreover, it was noted that in the samples before digestion and in the liquids at the individual steps of digestion, the content of the compounds identified in the breads containing microcapsules with inulin and maltodextrin was similar.

Zheng et al. (2018) observed during in vitro digestion of hawthorn fruit that (−)-epicatechin trimers and dimers were identified in the intestines (small and large) in very small amounts, and their greatest loss (over 50%) occurred between digestion in the mouth and stomach, i.e., at an earlier stage than in our own research on bread with the addition of microencapsulated hawthorn bark extract. Zhu et al. [59], while assessing the transformations of (−)-epicatechin monomers and dimers, noted that the chemical transformations of these compounds (degradation and isomerization) depend on the pH and both the acidic and alkaline environments accelerate these transformations. Perhaps, in our research, the encapsulation process delayed the transformations of these compounds and their greater loss occurred at a later stage of digestion. Other changes were observed by Ottaviani et al. [22] during in vivo digestion of (−)-epicatechin. They found that the absorption efficiency of (−)-epicatechin in the human body is approximately 80% and identified approximately 20 metabolites of (−)-epicatechin in blood and feces. In the light of these reports, attention is drawn to the difference in the level of complexity of (−)-epicatechin transformations under in vitro and in vivo digestion conditions.

Two bioactive compounds were identified in breads containing microcapsules with onion husk extract: quercetin and its glucoside (Table 3).

There were more of them in the samples containing microcapsules with maltodextrin than in the samples with the microencapsulated extract in inulin. During the digestion of the bread after the first and second step (digestion in the mouth and gastric digestion, respectively), the content of flavonols was traceable, and after the digestion of the enteric (step III) and in the membrane (step IV), none of the compounds present in the enriched onion extract were identified. Perez-Moral et al. [25], on the other hand, during the simulated digestion of heat-treated vegetables, identified quercetin at each stage of digestion and observed an increase in its content at individual stages. The reason for the differences between our results and the results of the cited authors may be the fact of different research material, in which there is a number of interactions between the macronutrients of cereals and plants and polyphenols [23].

#### 2.2.2. Antioxidant Capacity

Bioactive compounds are very sensitive to various environmental factors, which means that their properties may change significantly depending on the conditions in which they are found. For this reason, research is being undertaken on changes in antioxidant activity at various stages of digestion of products rich in such compounds or products enriched with them.

In breads enriched with hawthorn, onion and soybean polyphenols such as procyanidins, flavonols and isoflavones, the antioxidant activity (ABTS^+•^ and FRAP) was determined at various stages of simulated in vitro digestion. Due to the very low antioxidant activity in the control breads before digestion, the analysis of their antioxidant activity during in vitro digestion was omitted.

The results of the ability to reduce free radicals by the ABTS^+•^ method and Fe^2+^ ions by the FRAP method at individual stages of simulated in vitro digestion of breads enriched with microcapsules containing extracts of hawthorn bark, soybeans and onion husks are presented in Table 4 and Table 5.

It was found that the samples after the first stage of digestion had low antioxidant activity. ABTS^+•^ values ranged from 0.27 µmol TE/g DW (HtI bread) to 1.33 µmol TE/g DW (OM bread), and in the FRAP method in the range of 0.15 µmol TE/g DW (SM breads) to 1.32 µmol TE/g DW (HtI breads). The HtI (ABTS—1.50 and FRAP—0.13 µmol TE/g DW) and OI (ABTS—1.82 and FRAP—0.33 µmol TE/g DW) breads had the highest antioxidant activity of the absorbed compounds (membrane).

When assessing the changes in ABTS^+•^ radical scavenging activity after individual stages of digestion of the tested bread, it was found that in the liquids collected during the digestion of bread with HtM and HtI, the antiradical activity of ABTS^+•^ was the highest after the second and third step of in vitro digestion, and in the breads with the addition of SM and SI after step II and in samples from the membrane (stage IV) (Table 4). During the digestion of breads with the addition of onion husk extract, the influence of the carrier type on the ABTS^+•^ values was found because during the digestion of OI bread, the highest activity with the ABTS^+•^ method was found in the sample taken from the membrane (stage IV) and in the OM breads in the samples after the first and second stage.

Slightly different results of the antioxidant activity during the digestion of bread were obtained when assessing the ability to reduce Fe^+2^ ions using the FRAP method (Table 5). During the digestion of breads enriched with microencapsulated extracts obtained from soybean, hawthorn and onion, the ability to reduce iron ions was greatest at stages I and II, and it decreased at stage III (intestine), reaching the lowest level in samples taken from the membrane (stage IV). The highest ability to reduce iron ions was characterized by breads containing microcapsules with hawthorn extracts (HtM, HtI). Samples of this bread taken during digestion at the first and second stage were characterized by four times higher activity compared to bread samples with the addition of soybean isoflavones microcapsules (SM, SI) and four times higher than the bread with the addition of onion flavonols (OM, OI).

Moreover, it was observed that the activity of the compounds absorbed from the breads with the addition of hawthorn bark extract and onion husk (ABTS^+•^, FRAP) was higher when added in the form of microcapsules with inulin (HtI, OI) compared to the samples containing microcapsules with maltodextrin (HtM, OM) (Table 4 and Table 5). For soybean extracts, maltodextrin was the better carrier, as indicated by ABTS^+•^ values in samples taken from the membrane. The reason for the better effect of maltodextrin compared to inulin may be due to differences in the structure of these compounds and interactions between the extract compounds and the carrier. In maltodextrin there are mainly α-1,4-glycosidic bonds, while in inulin mainly β-2,1-glycosidic. This difference may account for the higher antioxidant activity in bakery products containing maltodextrin microcapsules at the first digestion stage because α-amylase (present in saliva) hydrolyzes only α-1,4-glycosidic bonds, i.e., those found in maltodextrin. Rodríguez-Roque et al. [26], examining the digestion process of soy milk, observed the highest antioxidant activity after the second stage (gastric digestion). Zheng et al. [21], on the other hand, during the simulated digestion of hawthorn fruit, found that the antioxidant activity was at the highest level after passing the last stage corresponding to digestion in the large intestine. Lai et al. [60] observed that the use of flavonols in combination with maltodextrin significantly increases their solubility—from 5.2% to 66.73%—which may also affect the results obtained in our research. The higher activity of maltodextrin-fixed breads may be due to the ability of the maltodextrin to remain in an amorphous form over a wide range of water activities. This property facilitates the growth of air bubbles during spray drying and there is more space for compounds with antioxidant properties in the spaces created by the growth of in vitro bubbles [49].

## 3. Materials and Methods

### 3.1. Chemicals

The compounds 6-hydroxy-2,5,7,8-tetramethylchroman-2-carboxylic acid (Trolox), 2,2′-azinobis-(3-ethylbenzothiazoline-6-sulfonic acid) (ABTS), formic acid, acetonitrile, pancreatin (8 × USP), pepsin (3200–4500 units/mg protein) and bile salts were obtained from Sigma-Aldrich (Steinheim, Germany). Acetone, anhydrous glucose, NaHSO_3_, sodium citrate, sodium benzoate, and potassium phosphate were purchased from Chempur (Piekary Slaskie, Poland). Acetonitrile LC-MS came from POCh (Gliwice, Poland), fructose from Biofan (Piekary Slaskie, Poland), potassium chloride and sodium chloride from STANLAB (Lublin, Poland). Daidzin and genistin were purchased from Extrasynthese (Genay, France). Inulin Orafti HPX (DP ≥ 23) was purchased from HORTIMEX PLUS (Konin, Poland). Maltodextrin (8 DE) came from the Department of Food Storage and Technology of Wrocław University of Environmental and Life Sciences.

### 3.2. Material

The research material consisted of wheat bread baked from commercial wheat flour, type 550, produced by Młyn Stradunia, GoodMills Polska Sp. z o.o. (wet gluten 34%, falling number 351 s) and microencapsulated in inulin (DP ≥ 23) or maltodextrin (DE = 8) extracts: from the bark of young shoots of hawthorn (*Crataegus monogyna* Jacq.)), from soybeans (*Glycine max* L.) and from onion husks (*Allium cepa* L.). The ratio of extract to carrier in the microcapsules was 1:3. The extract microcapsules were obtained at the Department of Fruit, Vegetable and Plant Nutraceutical Technology of Wrocław University of Environmental and Life Sciences.

### 3.3. Obtaining the Extract of Hawthorn, Soybeans and Onion Husks

The extraction procedure was performed according to the methodology described by Wyspiańska et al., [58]. The milled young shoots of hawthorn, soybeans and onion husks were treated with hexane for degreasing. Then a double extraction with the use of 80% ethanol was performed. After extraction, the samples were sonicated for 15 min and centrifuged (10 min, 3000 rpm). The obtained extracts were concentrated at 40°C under reduced pressure. Next the concentrates were applied to a column filled with Amberlite XAD 16 resin. The polyphenols were eluted with 80% ethanol, and the obtained ethanolic solutions were concentrated in a vacuum and then dried (39–40 °C, 0.094 MPa). The preparation yield was 0.8%.

### 3.4. Production of Microencapsulated Extracts

The microencapsulated extracts of hawthorn, soybeans and onion husks were obtained by spray-drying. Spray drying was performed according to Wyspiańska et al. [61]. Isoflavones from soybeans, procyanidins from hawthorn and flavonols from onion were suspended in water to give a 10% w/v dispersion [62]. To obtain a product to medium content in the ratio of 1:3 (w/w; polyphenols:carrier), inulin and maltodextrin were added in the appropriate proportions to the polyphenolic solutions. The solutions were homogenized (2 min) and spray dried in a mini spray dryer (BÜCHI, Flawil, Switzerland). The inlet air temperature was 150 °C and the raw material feed flow: 615 mL/hr. Temperature of the raw material was 40 °C.

### 3.5. Baking Procedure of Bread

The baking of wheat flour bread was carried out by the single-phase method. The basic bread formula consisted of wheat flour (50.00 g), salt (1.5% flour basis), and compressed yeast (3% flour basis). Microcapsules obtained from soybean, hawthorn and onion husks with the use of maltodextrin (SM, HtM, OM, respectively) and inulin (SI, HtI, OI, respectively) were added to the dough in the amount of 4% (flour basis), which corresponded to 1% of pure extract. Control samples were wheat bread without additives (WB), wheat bread with the addition of 3% (flour basis) inulin (WBI) or maltodextrin (WBM). The dough was made in a Farinograph mixer (Brabender GmbH & Co KG, Duisburg, Germany). The water to make the dough was added in the appropriate amount to obtain a consistency of 350 BU. The dough was fermented at 30 °C and 85% RH for 90 min (with 1 min transfixion after 60 and 90 min). Then the dough was placed in the molds (90 × 70 × 60 mm) and set aside for final fermentation (temperature 30°C, relative air humidity 85%, time about 42 min). The bread was baked in an electric oven GT 800 (IBIS, Szubin, Poland) (temperature 230 °C, time 20 min, chamber initial steaming—4 min).

### 3.6. Determination of Bread Yield, Volume, Color and Storage Conditions

The baked breads were cooled for approximately 1 h to ambient temperature and then weighed and the volume was measured. Based on the weight of the obtained bread, the bread efficiency was calculated in relation to the weight of flour used for its baking. The volume of bread was determined using poppy seeds. The cylindrical container was filled with poppy seeds and its surface was smoothed. The volume of poppy seeds measured in this way was poured into the container and the surface was leveled. The volume of the scraped off excess poppy seeds, equivalent to the volume of the bread, was measured in a measuring cylinder (500 cm^3^). The measurement was performed in duplicate (the difference between the replicates was not greater than 5%). The determined volume and weight of the loaf of bread was used to calculate the specific volume of bread. The color of the bread crumb was determined in the CIE L* a* b* system using a Konica Minolta Chroma Meter CR-2000b colorimeter (Ramsey, USA).

### 3.7. Preparation of Bread Crumb Extracts for HPLC and Antioxidant Activity Determinations

The bread was cut into slices about 10 mm thick and the crusts were cut from them. The crumb was placed in a F618AW freezer (Gorenje, Velenje, Slovenia) and stored at −18 °C for 24 h. It was then freeze-dried for 10 h in a 1–4 LSC freeze dryer (ChristAlpha, Osterode am Harz, Germany). The average moisture content of the lyophilisates was 3%. The freeze-dried crumb was ground in a laboratory mill WŻ-1 (Zakład Badawczy Przemysłu Piekarskiego Sp. z o.o., Bydgoszcz Poland) and stored at −80 °C (Kaltis 499, Taipei, Taiwan). The test tubes were weighed with 1 g (with an accuracy of 0.001 g) of crushed, freeze-dried bread crumb and 5 mL of 80% HCl methanol (1 mL of 36% HCl per 1 L of 80% methanol) was added. The samples were sonicated for 15 min in a Sonic 6D ultrasonic bath (Polsonic, Warsaw, Poland) and then shaken (10 min, 150 rpm) on a GFL 1092 orbital shaker (Burgwedel, Germany). After shaking, the samples were centrifuged (10 min, 3000 rpm) in an MPV-370 centrifuge (POCH, Gliwice, Poland). The supernatant solution was poured into a 5 mL volumetric flask and made up to 5 mL with 80% methanol with HCl, and then transferred to 15 mL test tube. Four mL of 80% methanol with HCl was added to the centrifugation pellet and sonicated, shaken and vortexed a second time. The solution was poured into 5 mL flasks and made up to 5 mL with 80% methanol with HCl and poured into the test tubes containing the first fraction of extracts. The combined solutions were mixed and centrifuged (5 min, 10,000 rpm) in an MPV 350R centrifuge (POCH, Gliwice, Poland). In this way, 10 mL of methanol extracts were obtained from 1.0000 g of dry weight of breads.

### 3.8. Determination of Polyphenols by HPLC

The content of polyphenols was determined by high-performance liquid chromatography HPLC on a Dionex chromatograph (Sunnyvale, CA, USA). The chromatograph was equipped with an Ultimate 3000 diode detector, LPG-3400A pump, EWPS-3000SI autosampler, TCC-3000SD column thermostat and Chromeleon v.6.8 software (Thermo Fisher Scientific, Waltham, MA, USA). The method was previously described by Kucharska et al. [63]. Separation of compounds was obtained using a Cadenza C18 column (75 × 4.6 mm) with a solution of 4.5% formic acid (A) and 100% acetonitrile (B). Separation was obtained using a gradient: 0–1 min 5% B, 20 min 25% B, 21 min 100% B, 26 min 100% B, 27 min 5% B. Flow rate 1 mL/min. The column was thermostated at 30 °C. Flavonols were monitored at 360 nm, procyanidins and isoflavones at 280 nm and anthocyanins at 520 nm. The content of flavonols was converted to quercetin, procyanidins to (–)-epicatechin, isoflavones to daidzein, glycitin and genistin glycosides [61].

### 3.9. Determination of Antioxidant Activity by ABTS^+•^ Method

The antioxidant activity based on the inactivation of the radical ABTS^+•^ was determined by the method described by Re et al. [64]; 0.03 mL of the diluted (×2–×20 dilutions were used) and 3 mL of ABTS^+•^ ethanol solution were dispensed into cuvettes. After 6 min, the absorbance at λ = 734 nm was measured on a Shimadzu UV-2401 PC spectrophotometer (Kyoto, Japan) against blank water. The antioxidant activity was expressed in μmol Trolox per g dry weight (μmol Trolox/g DW). The analysis was performed in triplicate for each sample.

### 3.10. Determination of Iron Ion Reduction Force Using FRAP Method

The reduction force of Fe^2+^ ions was determined using the FRAP method [65]. One mL of diluted (×20 dilutions were used) sample and 3 mL of methanolic FRAP solution were dispensed into cuvettes. After 10 min, the absorbance was measured on a Shimadzu UV-2401 PC spectrophotometer (Kyoto, Japan) at a wavelength of λ = 593 nm against water as a blank. The antioxidant activity was expressed in μmol Trolox per g dry weight (μmol TE/g DW). The test was performed in triplicate for each sample.

### 3.11. In Vitro Digestion

The simulated in vitro digestion was carried out according to the methodology described by Gawlik-Dziki [66]. The first stage of digestion (I) is simulated digestion by oral α-amylase. The next stage (II) is digestion in the stomach—the action of pepsin in a strongly acidic environment (pH = 1.2). The third stage (III) reflects intestinal digestion with the addition of pancreatin and bile extract in an environment with increased pH. Compounds absorbed during digestion were determined in samples taken from the membrane (IV).

In vitro digestion was carried out in triplicate in 250 mL glass beakers covered with parafilm. Ten g of the sample was placed in a beaker and 30 mL of stimulated saliva was added to the mixture of Na_2_HPO_4_, KH_2_PO_4_, NaCl and E.C. 3.2.1.1. α-amylase with an activity of 200 U/mL, then placed in a shaker water bath at 37 °C for 10 min (step I—simulated digestion by oral α-amylase). After incubation, 5 mL samples were taken for analysis of antioxidant activity and polyphenol content. The remaining solution was subjected to further gastric digestion (step II—treatment with pepsin in a strongly acidic environment (pH = 1.2)). The sample in the beaker was adjusted to pH = 1.2 with 6 M HCl, and then 30 mL of gastric fluid (0.32% pepsin solution (Sigma, product number P6887-1G) in 0.03 M NaCl at pH = 1) was added to it. The beaker was placed in a 37 °C water bath with shaking for 120 min. At the end of incubation, 5 mL was taken from each sample. In stage III of digestion (intestinal digestion with the addition of pancreatin and bile extract in an environment with increased pH), the remaining sample after stage II was adjusted to pH = 5.0 with 0.5 M NaHCO_3_ and the amount of 0.5 M NaHCO_3_ should be added to the sample, to adjust the solution to pH = 7.5. The dialysis membranes (stage IV of digestion) were filled with a calculated amount of 0.5 M NaHCO_3_. After the membranes were filled and sealed, they were placed in beakers so that they were completely immersed in the solution, and then the beakers were placed in a 37 °C water bath with shaking for 30 min. After this time, 30 mL of the mixture prepared from 0.05 g of pancreatin (Sigma, product number P7545) and 0.3 g of bile extract (Sigma, B8756-10G`1), dissolved in 35 mL of 0.1 M NaHCO_3_, were added to the beaker and placed back in a water bath with shaking for 90 min. After this time, 5 mL samples were taken from the solution in the beaker (compounds remaining after enteric digestion) and from the center of the dialysis membranes (compounds absorbed during digestion). At all stages of digestion, the qualitative and quantitative changes of the active compounds were determined by HPLC and the antioxidant activity was determined by ABTS^+•^ and FRAP methods. For this purpose, the samples were centrifuged, diluted and purified on Whatman filters (0.45 µm). Prior to the determination of the antioxidant activity, the samples were purified on Sep-Pak C18 mini-columns. The content of polyphenols was expressed as mg per 1 mL of samples (mg/mL) and the antioxidant activity was expressed as μmol Trolox per mL of sample (μmol TE/mL).

### 3.12. Statistical Analysis

The results were analyzed statistically by Statistica 13.1 software (StatSoft, Kraków, Poland), using one-way analysis of variance (ANOVA). Differences were rated by Duncan’s test at the significance level α = 0.05.

## 4. Conclusions

Based on the results obtained in samples, it can be concluded that the addition of microencapsulated extracts to wheat bread significantly increased the content of polyphenols and antioxidant activity. The richest source of polyphenolic compounds among the studied raw materials was soybean, while the addition of hawthorn caused the smallest changes in the concentration of these compounds. Microcapsules of soy extracts only slightly influenced the volume and color of the bread, and the onion husk preparations significantly reduced the volume and darkened the color. Breads with microcapsules in which maltodextrin was the carrier were characterized by a higher polyphenol content than breads with inulin protected extracts. A reverse relationship was observed in breads with onion husk extract and no significant differences were found in the breads with the addition of hawthorn extract due to the carrier of the extract. The greatest ability to scavenge ABTS radicals was demonstrated in breads containing onion husk extract, and those with hawthorn bark extract showed the greatest ability to reduce Fe ions. Breads with soybean extract had the lowest antioxidant activity. No differences in antioxidant activity were found between breads containing microcapsules with different carriers. During in vitro digestion, it was found that the procyanidins and isoflavones in bread were more resistant to digestive processes than flavonols. The content of polyphenols successively decreased at consecutive stages of digestion, and the antioxidant activity was highest at the digestive stage in the oral cavity (I) and stomach (II). The results may suggest that the process of microencapsulation increased bioavailability of the polyphenols in the gastrointestinal tract. Due to the fact that in breads enriched with plant extracts, the content of identified polyphenols and antioxidant activity during the simulated in vitro digestion changed in a very different way, depending on the type of extract and the type of carrier in the microcapsules, it cannot be indicated which of the used carriers had a better protective effect on polyphenols. This issue requires further study.

## Figures and Tables

**Table 1:** Concentration on polyphenolic compounds (mg/mL) of liquids released during in vitro digestion of wheat bread enriched with soy extracts (SI, SM).

Carrier of Extract	In Vitro Digestion Step	Isoflavones (mg/mL) *									
		Daidzin	Glycitin	Genistin	Malonyl-daidzin	Malonyl-glycitin	Malonyl-genistin Isomer	Malonyl-genistein	Daidzein	Genistein	Total
Maltodextrin	I	0.23	nd	0.40	0.33	0.08	0.39	0.47	nd	0.35	2.26 a
	II	0.14	nd	0.34	0.23	nd	0.32	0.48	nd	0.31	1.83 b
	III	0.08	nd	0.19	0.17	nd	0.28	0.34	nd	0.30	1.37 c
	IV	0.06	nd	0.11	0.08	0.01	0.09	0.16	nd	0.12	0.62 d
Inulin	I	0.14	nd	0.25	0.38	0.08	0.42	0.32	nd	0.21	1.79 a
	II	0.13	nd	0.24	0.18	nd	0.24	0.43	nd	0.36	1.57 b
	III	0.06	nd	0.12	0.14	nd	0.20	0.29	nd	0.29	1.10 c
	IV	0.04	nd	0.07	0.10	0.01	0.11	0.16	nd	0.13	0.62 d

* Values with different letters were significantly different at *p* = 0.05 according to Duncan’s test. Values were converted into daidzein (daidzin and malonyldaidzin) or genistein (glycitin, genistin, malonylglycitin, malonylgenistin isomer and malonylgenistein). Abbreviations: I—liquid after simulated saliva digestion; II—liquid after gastric fluid digestion; III—liquid after simulated intestinal fluid digestion; IV—liquid after membrane; nd—not detected.

**Table 2:** Concentration on polyphenolic compounds (mg/mL) of liquids released during in vitro digestion of wheat bread enriched with hawthorn extracts (HtM, HtI).

Carrier of Extract	In Vitro Digestion Step	Procyanidins (mg/mL) *								
		Procyanidin Dimer B2	Procyanidin Dimer B6	(−)-Epicatechin	Procyanidin Trimer C1	Procyanidin Tetramer I	Procyanidin Tetramer II	Procyanidin Trimer	Procyanidin Tetramer III	Total
Maltodextrin	I	0.06	0.02	0.53	0.53	0.02	0.02	nd	nd	1.18 a
	II	0.05	0.03	0.3	0.28	0.06	0.01	nd	0.03	0.75 b
	III	nd	nd	0.3	0.02	nd	nd	nd	nd	0.32 c
	IV	nd	nd	0.11	nd	nd	nd	nd	nd	0.11 d
Inulin	I	0.06	0.02	0.43	0.52	0.03	0.02	nd	nd	1.09 a
	II	0.05	0.04	0.39	0.35	0.07	0.01	0.01	0.04	0.97 b
	III	nd	nd	0.3	0.02	nd	nd	nd	nd	0.32 c
	IV	nd	nd	0.11	nd	nd	nd	nd	nd	0.11 d

* Values with different letters were significantly different at *p* = 0.05 according to Duncan’s test. Values were converted into (−)-epicatechin. Abbreviations: I—liquid after simulated saliva digestion; II—liquid after gastric fluid digestion; III —liquid after simulated intestinal fluid digestion; IV—liquid after membrane; nd—not detected.

**Table 3:** Concentration on polyphenolic compounds (mg/mL) of liquids released during in vitro digestion of wheat bread enriched with onion extracts (OM, OI).

Carrier of Extract	In Vitro Digestion Step	Flavonols (mg/mL) *						
		Quercetin 7,4-diglucoside	Quercetin 3,4-diglucoside	Quercetin 3-glucoside	Quercetin 4-glucoside	Isorhamnetin 4-glucoside	Quercetin	Total
Maltodextrin	I	nd	nd	nd	nd	nd	0.01	0.01 a
	II	nd	nd	nd	0.01	nd	0.03	0.03 a
	III	nd	nd	nd	nd	nd	nd	nd
	IV	nd	nd	nd	nd	nd	nd	nd
Inulin	I	nd	nd	nd	nd	nd	0.01	0.01 a
	II	nd	nd	nd	nd	nd	0.02	0.02 a
	III	nd	nd	nd	nd	nd	nd	nd
	IV	nd	nd	nd	nd	nd	nd	nd

* Values with different letters were significantly different at *p* = 0.05 according to Duncan’s test. Values were converted into quercetin. Abbreviations: I—liquid after simulated saliva digestion; II—liquid after gastric fluid digestion; III—liquid after simulated intestinal fluid digestion; IV—liquid after membrane; nd—not detected.

**Table 4:** ABTS antioxidant capacity (μmol TE/mL D.W.) of liquids released during in vitro digestion of wheat bread enriched with different extracts.

Carrier of Extract	In Vitro Digestion Step	ABTS (μmol TE/mL D.W)		
		Soy	Hawthorn	Onion
Maltodextrin	I	0.51 ± 0.12 b	0.69 ± 0.08 c	1.33 ± 0.18 a
	II	0.99 ± 0.06 a	2.60 ± 0.17 a	1.49 ± 0.15 a
	III	0.47 ± 0.13 b	2.87 ± 0.14 a	1.16 ± 0.10 b
	IV	0.83 ± 0.03 a	1.04 ± 0.10 b	0.32 ± 0.17 c
Inulin	I	0.31 ± 0.07 c	0.27 ± 0.10 d	0.43 ± 0.07 c
	II	0.69 ± 0.07 a	3.19 ± 0.19 a	1.62 ± 0.09 b
	III	0.49 ± 0.07 b	2.29 ± 0.24 b	1.50 ± 0.16 b
	IV	0.61 ± 0.02 a	1.50 ± 0.13 c	1.82 ± 0.19 a

* Values with different letters were significantly different at *p* = 0.05 according to Duncan’s test. The data are expressed as mean ± SD (n = 3). Abbreviations: I—liquid after simulated saliva digestion; II—liquid after gastric fluid digestion; III—liquid after simulated intestinal fluid digestion; IV—liquid after membrane.

**Table 5:** FRAP antioxidant capacity (μmol TE/mL D.W.) of liquids released during in vitro digestion of wheat bread enriched with different extracts.

Carrier of Extract	In Vitro Digestion Step	FRAP (μmol TE/mL D.W)		
		Soy	Hawthorn	Onion
Maltodextrin	I	0.15 ± 0.01 a	1.43 ± 0.06 a	0.40 ± 0.02 a
	II	0.13 ± 0.02 a	1.16 ± 0.06 a	0.49 ± 0.03 a
	III	0.09 ± 0.02 a	0.50 ± 0.05 b	0.33 ± 0.02 b
	IV	0.03 ± 0.01 b	0.21 ± 0.02 c	0.03 ± 0.01 c
Inulin	I	0.16 ± 0.02 a	1.32 ± 0.08 a	0.38 ± 0.04 a
	II	0.13 ± 0.01 a	1.13 ± 0.02 a	0.37 ± 0.04 a
	III	0.06 ± 0.02 b	0.58 ± 0.07 b	0.27 ± 0.02 b
	IV	0.01 ± 0.01 b	0.13 ± 0.08 c	0.33 ± 0.02 b

* Values with different letters were significantly different at *p* = 0.05 according to Duncan’s test. The data are expressed as mean ± SD (*n* = 3). Abbreviations: I—liquid after simulated saliva digestion; II—liquid after gastric fluid digestion; III—liquid after simulated intestinal fluid digestion; IV—liquid after membrane.

## Data Availability

Data is contained within the article.

## Supplementary Materials

The following are available online at. Table S1: Color of different samples of wheat bread, Table S2: Characteristics of soy extracts microcapsules and bread with addition of microcapsules of soy extracts encapsulated with inulin and maltodextrin, Table S3: Characteristics of hawthorn extracts microcapsules and bread with addition of microcapsules of hawthorn extracts encapsulated with inulin and maltodextrin, Table S4: Characteristics of onion extracts microcapsules and bread with addition of microcapsules of onion extracts encapsulated with inulin and maltodextrin.

## Sample Availability

Samples of the compounds are not available from the authors.
