# Supplementary material for: Changes in Antioxidant Properties and Amounts of Bioactive Compounds during Simulated In Vitro Digestion of Wheat Bread Enriched with Plant Extracts
Source: Molecules. 2021 Oct 18;26(20):6292. doi: 10.3390/molecules26206292 (PMC8539602; doi:10.3390/molecules26206292)
Supplement: Supplementary file 1 [file molecules-26-06292-s001.zip › molecules-1382027-supplementary.pdf]

Supplementary data

Table S1. Color of different samples of wheat bread.

Colour parameter	WB	Maltodextrin				Inulin			
		WBM	SM	HtM	OM	WBI	SI	HtI	OI
L*	66,20	59,60	58,80	49,00	34,70	58,40	56,50	48,10	32,60
a*	-2,00	2,40	3,50	4,20	8,80	2,30	4,00	4,80	9,20
b*	11,30	14,40	16,70	9,40	8,60	14,10	17,00	8,90	7,60

* Abbreviations: WB – wheat bread; WBM – wheat bread with maltodextrin; SM – wheat bread enriched with soy extracts in maltodextrin; HtM – wheat bread enriched with hawthorn extracts in maltodextrin; OM – wheat bread enriched with onion husk extracts in maltodextrin; WBI – wheat bread with inulin; SI – wheat bread enriched with soy extracts in inulin; HtI – wheat bread enriched with hawthorn extracts in inulin; OI – wheat bread enriched with onion husk extracts in inulin. The data are expressed as mean (n = 3).

Table S2. Characteristics of soy extracts microcapsules and bread with addition of microcapsules of soy extracts encapsulated with inulin and maltodextrin

		Maltodextrin		Inulin	
		Microcapsules	Bread with microcpasules	Microcapsules	Bread with microcpasules
Isoflavones content [mg/g D.W.]	Daidzin	38.2	0.72	30.6	0.88
	Glycitin	5.1	0.14	4.1	0.22
	Genistin	54.3	2.3	45.1	2.57
	Malonyldaidzin	26.8	1.31	21.6	2.42
	Malonylglycitin	5.5	0.26	4.6	0.46
	Malonylgenistin isomer	4.7	1.91	3.7	0.35
	Malonylgenistein	43.0	3.85	35.1	3.06
	Daidzein	9.9	4.39	8.8	4.6
	Genistein	11.9	3.84	11.6	8.11
	Sum of polyphenols	199.4	18.17	165.5	22.67
Antioxidant activity [µmol TE/g D.W.]	$ABTS^{+\bullet}$	319	28.16	298	27.51
	FRAP	565	17.57	383	12.07

Table S3. Characteristics of hawthorn extracts microcapsules and bread with addition of microcapsules of hawthorn extracts encapsulated with inulin and maltodextrin

		Maltodextrin		Inulin	
		Microcapsules	Bread with microcpasules	Microcapsules	Bread with microcpasules
Procyanidin content [mg/g D.W.]	Procyanidin dimer B4	44.0	nd	42.2	nd
	Procyanidin dimer B5	25.6	0.16	22.0	0.18
	Procyanidin dimer B2	179.6	0.1	170.8	0.11
	(–)-Epicatechin	253.2	0.72	241.6	0.77
	Procyanidin trimer C1	109.2	1.13	105.2	1.17
	Procyanidin tetramer I	55.6	0.3	52.8	0.32
	Procyanidin tetramer II	44.4	0.06	43.6	0.06
	Procyanidin trimer	10.0	0.04	8.4	0.04
	Procyanidin tetramer III	29.2	0.1	27.2	0.12
	Sum of polyphenols	750.8	2.52	713.6	2.65
Antioxidant activity [µmol TE/g D.W.]	$ABTS^{+\bullet}$	2823	112.74	2768	120.27
	FRAP	1129	23.58	1082	16.47

Table S4. Characteristics of onion extracts microcapsules and bread with addition of microcapsules of onion extracts encapsulated with inulin and maltodextrin

		Maltodextrin		Inulin	
		Microcapsules	Bread with microcapsules	Microcapsules	Bread with microcapsules
Flavonols content [mg/g D.W.]	Quercetin 7,4-diglucoside	1.7	nd	2.0	nd
	Quercetin 3,4-diglucoside	2.0	nd	2.1	nd
	Quercetin 3-glucoside	1.6	nd	2.5	nd
	Quercetin 4-glucoside	122.8	0.76	133.3	0.54
	Isorhamnetin 4-glucoside	4.7	nd	5.3	nd
	Quercetin	792.2	17.59	880.1	15.65
	Sum of polyphenols	925.0	18.35	1025.2	16.9
Antioxidant activity [μmol TE/g D.W.]	$ABTS^{+\bullet}$	525	133.02	331	125.99
	FRAP	627	17.5	440	12.98
